# Supplementary figures and images for: Polarized lung inflammation and Tie2/angiopoietin-mediated endothelial dysfunction during severe Orientia tsutsugamushi infection
Source: PLoS Negl Trop Dis. 2020 Mar 2;14(3):e0007675. doi: 10.1371/journal.pntd.0007675 (PMC7067486; doi:10.1371/journal.pntd.0007675)

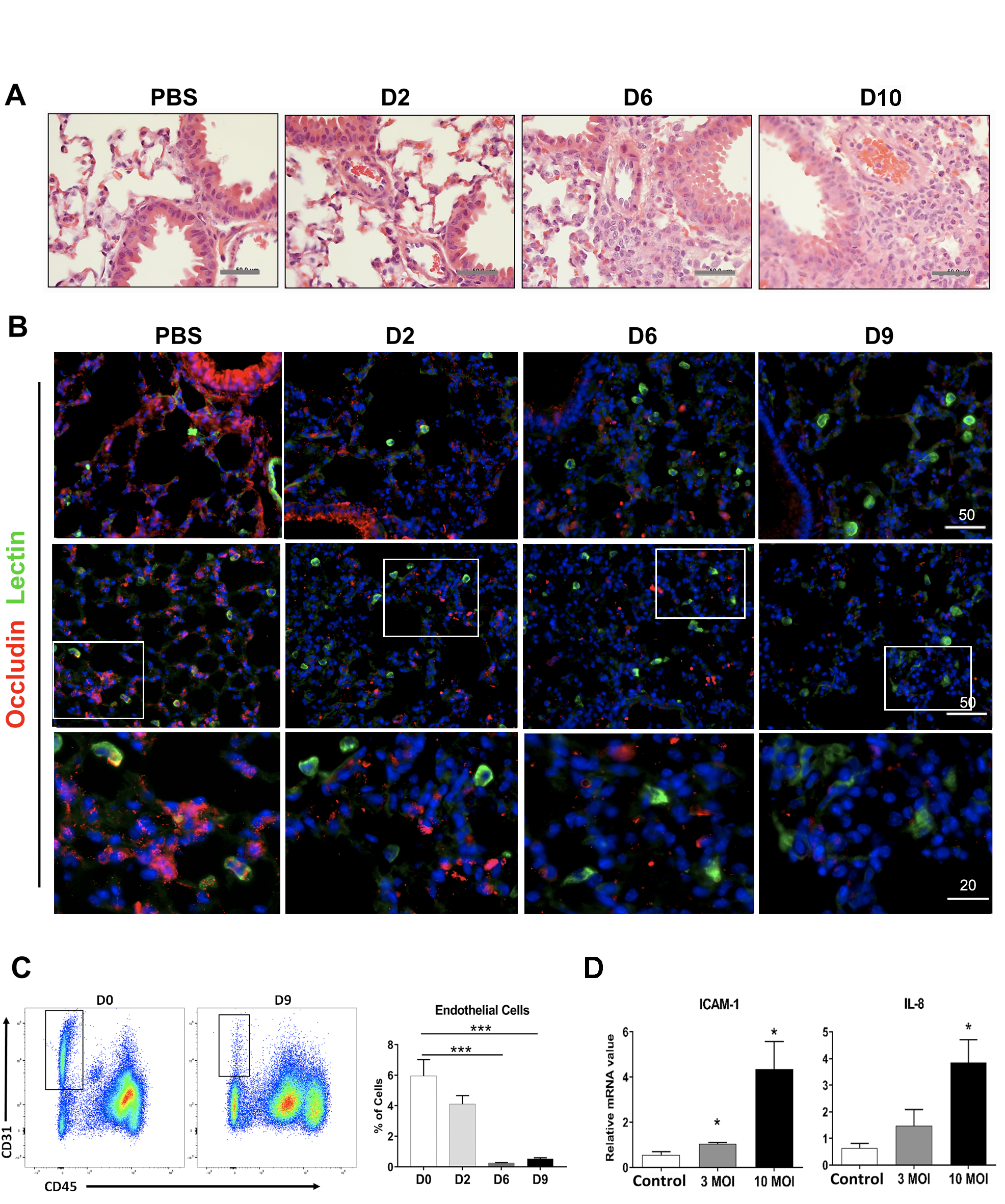

Supplement: S1 Fig — Female C57BL/6J mice (4–6 mice/group) were inoculated with 1.325 x 10^6^ of O. tsutsugamushi Karp strain. At indicated days of infection, equivalent lung portions were collected. (A) Hematoxylin and eosin staining of lung tissues during lethal challenge demonstrating increased cellular infiltration and alveolar thickening as the infection progresses (scale bars = 50 μm). (B) Frozen sections were processed for immunofluorescent staining and co-stained for occludin (cell-cell tight junctions, red), FITC-labeled GSL I-B4 lectins (green, top rows, scale bars = 50 μm), and DAPI (blue). The close-up views of the boxed areas are shown in the lower row (bar = 20 μm). (C) Flow cytometry analysis of viable pulmonary ECs (CD31+CD45-) collected at early (D0) and late (D9) infection. (D) Cultured HUVECs were infected with bacteria at 3 or 10 multiplicity of infection (MOI, 4 samples/group) and analyzed via qRT-PCR for gene expression at 24 h post-infection. Data are presented as relative to GAPDH values. *, p < 0.05; **, p < 0.01; and ***, p < 0.001 compared to PBS controls. Graphs are shown as mean +/- SEM. Flow cytometric and qRT-PCR data were analyzed by using one-way ANOVA with Tukey’s Post Hoc. At least 3 independent mouse infection experiments and 2 independent in vitro experiments were performed with similar trends; shown are representative data. (TIF) [file pntd.0007675.s001.tif]

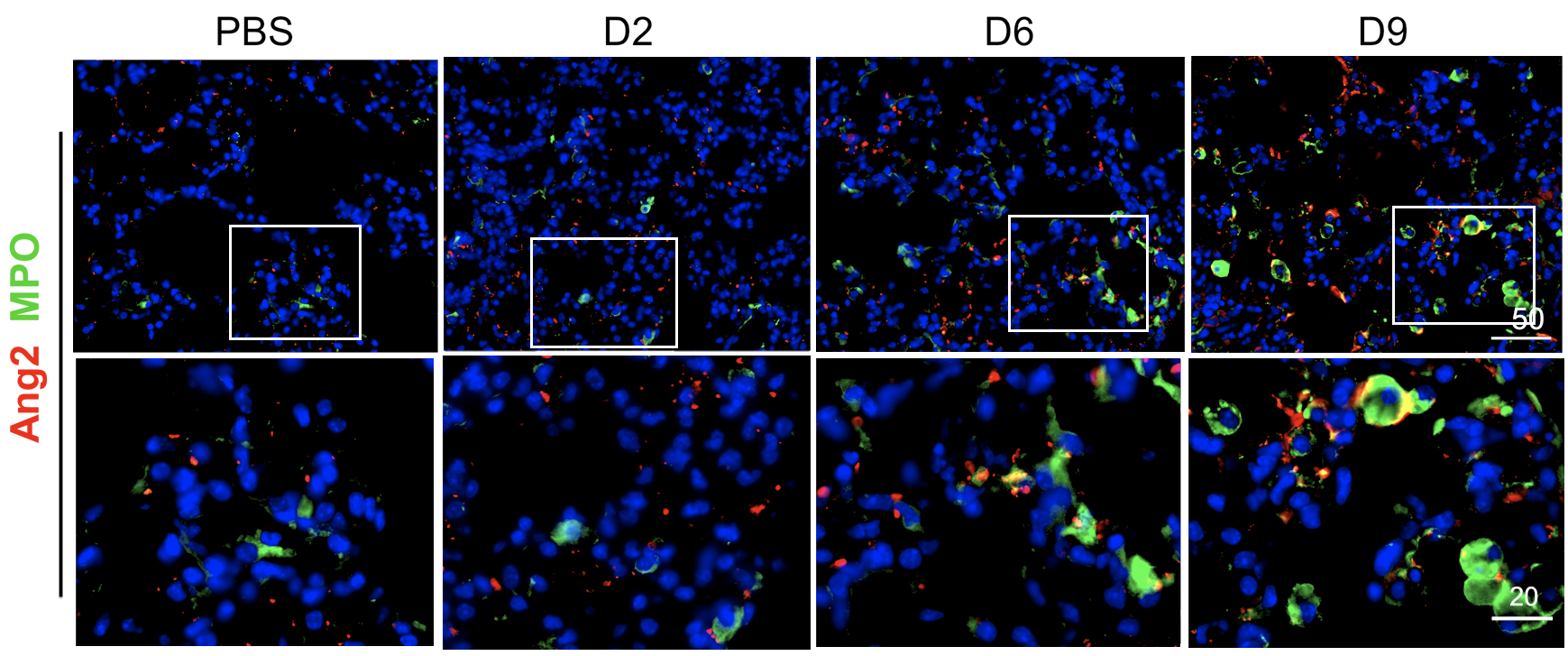

Supplement: S2 Fig — Mice were infected, and lung tissues were prepared for immunofluorescent analyses, as in Fig 1. Lung frozen sections were co-stained for MPO (green) and Ang2 (red). The low-magnification images (top rows, scale bar = 50 μm) and close-up views of the boxed areas (bottom rows, bar = 20 μm) are shown. (TIF) [file pntd.0007675.s002.tif]

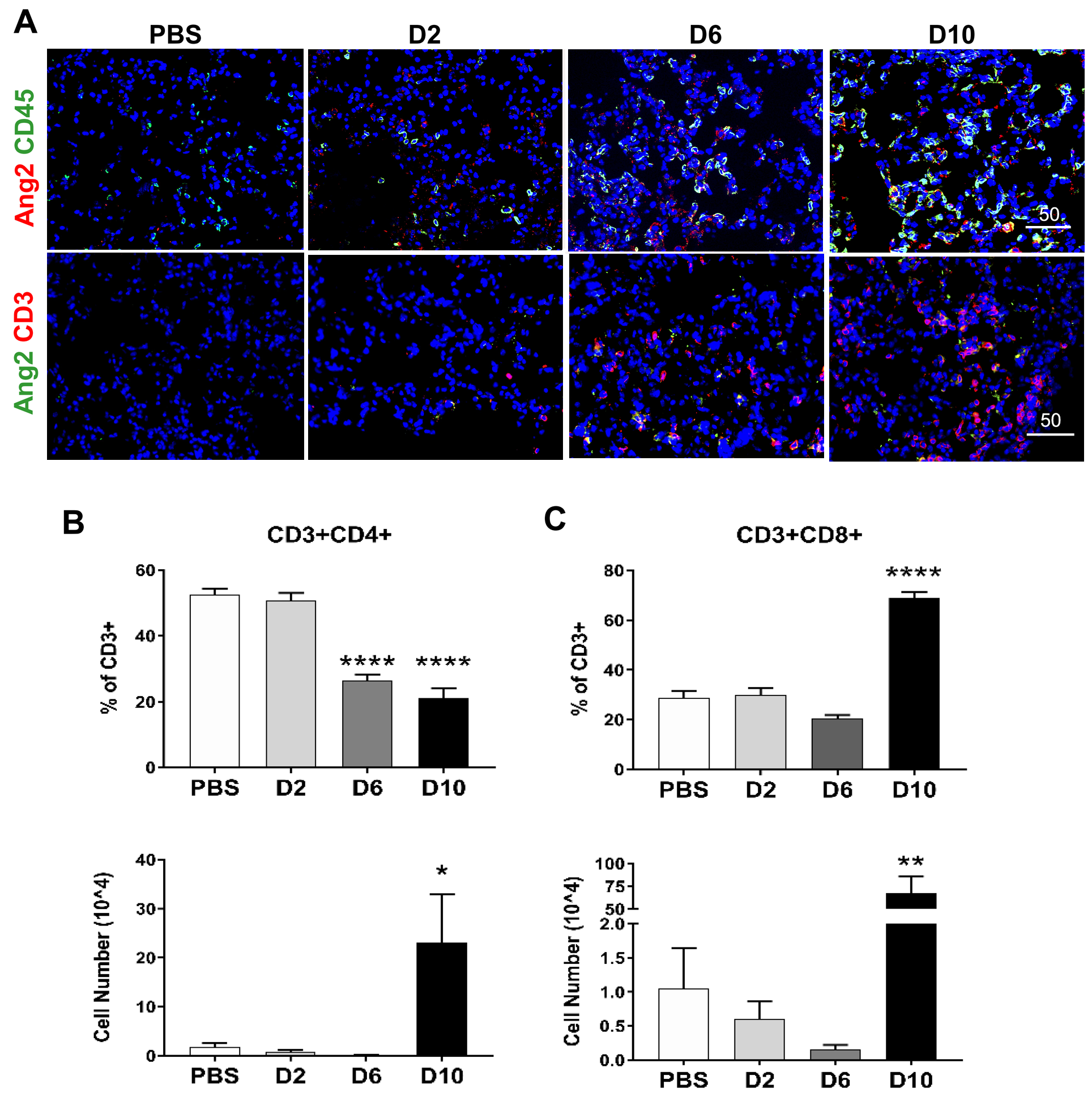

Supplement: S3 Fig — Female C57BL/6J mice (3–5 mice per group) were inoculated with 1.325 x 10^6^ of O. tsutsugamushi Karp strain. At indicated days of infection, equivalent lung portions were collected and processed for immunofluorescent staining or flow cytometric analysis. (A) Frozen sections were either co-stained for Ang2 (red) and CD45 (a leukocyte marker, green), or Ang2 (green) and CD3 (a T cell marker, red, bars = 50 μm). The percentage and absolute number of CD3+CD4+ T cells (B), as well as CD3+CD8+ T cells (C), were quantified and compared to non-infected controls (*, p<0.05; **, p<0.01; ****, p <0.0001). Graphs are shown as +/- SEM. Flow cytometry groups were analyzed using one-way ANOVA with Tukey’s Post Hoc. (TIF) [file pntd.0007675.s003.tif]

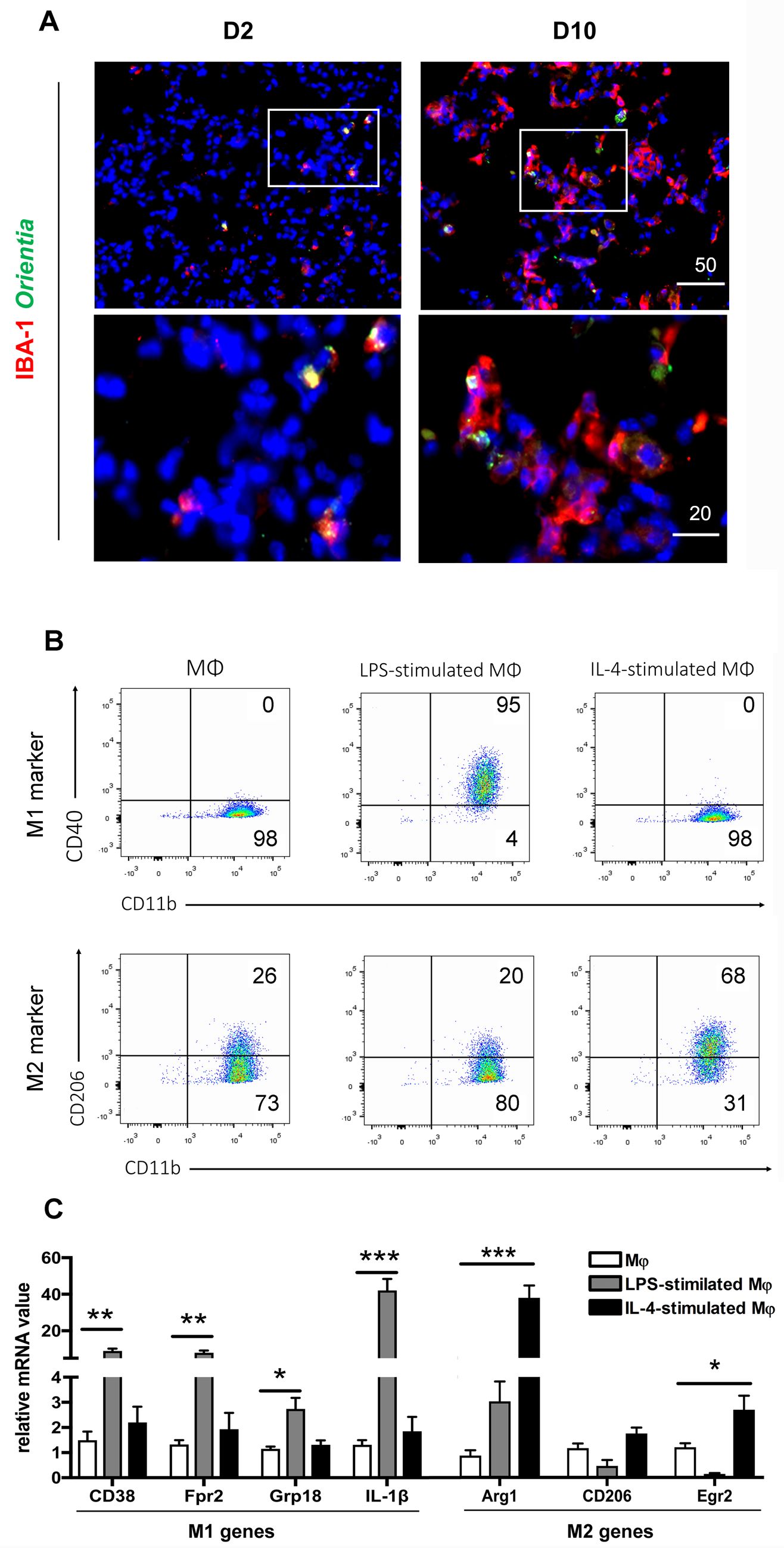

Supplement: S4 Fig — (A) Female C57BL/6J mice (4–6 mice per group) were inoculated with 1.325 x 10^6^ of O. tsutsugamushi Karp strain. At days 2 and 10, equivalent lung portions were processed; frozen sections were co-stained for Orientia (red), IBA-1 (green, a macrophage marker), and DAPI (blue), showing images in a low-magnification (top rows, scale bar = 50 μm) and close-up views of the boxed areas (bottom rows, bar = 20 μm). (B) Bone marrow-derived MΦs were treated with LPS (100 ng/ml) or rIL-4 (10 ng/ml) for 24 h and analyzed for the expression of indicated markers via flow cytometry. The numbers represent the percentages (%) of gated cells. (C) LPS- and IL-4-primed cells were analyzed by qRT-PCR for the expression of the indicated markers, showing the polarization of MΦ subsets compared with control cells [45, 74] (*, p < 0.05; **, p < 0.01; and ****, p < 0.0001). Data are shown as +/- SEM and were analyzed using one-way ANOVA with Tukey’s Post Hoc. (TIF) [file pntd.0007675.s004.tif]
