# Supplementary material for: Polarized lung inflammation and Tie2/angiopoietin-mediated endothelial dysfunction during severe Orientia tsutsugamushi infection
Source: PLoS Negl Trop Dis. 2020 Mar 2;14(3):e0007675. doi: 10.1371/journal.pntd.0007675 (PMC7067486; doi:10.1371/journal.pntd.0007675)
Supplement: S1 Table — The primer sequences used in this study (listed in the 5’ to 3’ direction). (PDF) [file pntd.0007675.s005.pdf]

S1 Table. Real-time PCR primers of tested genes.

Species	Gene	Forward	Reverse
**Human**	GAPDH	5’-ACAACTTTGGTATCGTGGAAGG-3’	5’- GCCATCACGCCACAGTTTC-3’
	ICAM-1	5’-ATGCCCAGACATCTGTGTCC-3’	5’-GGGGTCTCTATGCCCAACAA-3’
	IL-8	5’-TTTTGCCAAGGAGTGCTAAAGA	5’-AACCCTCTGCACCCAGTTTTC-3’
**Mouse**	β-actin	5’-CGAGGCCCAGAGCAAGAGAG-3’	5’-CGGTTGGCCTTAGGGTTCAG-3’
	Fpr2	5’-GAGCCTGGCTAGGAAGGTG-3’	5’-TCGTGAAACCAATAAGGAACCTG-3’
	CD38	5’-TCTCTAGGAAAGCCCAGATCG-3’	5’-GTCCACACCAGGAGTGAGC-3’
	NOS2	5’-GTTCTCAGCCCAACAATACAAGA-3’	5’-GTGGACGGGTCGATGTCAC-3’
	IFN-γ	5’-AACGCTACACACTGCATCTTGG-3’	5’-GCCGTGGCAGTAACAGCC-3’
	CD206	5’-CTCTGTTCAGCTATTGGACGC-3’	5’-CGGAATTTCTGGGATTCAGCTTC-3’
	Egr2	5’-GCCAAGGCCGTAGACAAAATC-3’	5’-CCACTCCGTTCATCTGGTCA-3’
	Arg1	5’-CTCCAAGCCAAAGTCCTTAGAG-3’	5’-AGGAGCTGTCATTAGGGACATC-3’
	IL-10	5’-GCTCTTACTGACTGGCATGAG-3’	5’-CGCAGCTCTAGGAGCATGTG-3’
**Bacteria**	*Orientia* 47-kDa	5’-AACTGATTTTATTCAAACTAATGCTGCT-3’	5’-TATGCCTGAGTAAGATACTGTAATGGA-3’
