# Supplementary material for: Polarized lung inflammation and Tie2/angiopoietin-mediated endothelial dysfunction during severe Orientia tsutsugamushi infection
Source: PLoS Negl Trop Dis. 2020 Mar 2;14(3):e0007675. doi: 10.1371/journal.pntd.0007675 (PMC7067486; doi:10.1371/journal.pntd.0007675)
Supplement: Attachment — Submitted filename: PNTD-D-19-01266_reviewer comments.docx [file pntd.0007675.s006.docx]

PNTD-D-19-01266_reviewer – reviewer comments

Minor comments

- Line 152 – The abbreviation of i.v should first appear here than in the line 418.
- Line 545- South Korea is an endemic country for scrub typhus and the baseline titers varies from region to region. Previously, the single cut off titers used have ranged from 1:10 to 1:400. How did the authors decide on ≥1:160 is a sufficient cut off titer on single serum/plasma specimen? It would be apt to cite a reference here.
- Line 548 – healthy volunteer’s sera was negative at 1:160 dilution?
