# Supplementary material for: Polarized lung inflammation and Tie2/angiopoietin-mediated endothelial dysfunction during severe Orientia tsutsugamushi infection
Source: PLoS Negl Trop Dis. 2020 Mar 2;14(3):e0007675. doi: 10.1371/journal.pntd.0007675 (PMC7067486; doi:10.1371/journal.pntd.0007675)
Supplement: Attachment — Submitted filename: PNTD-D-19-01266_Resposnes to Reviewers.docx [file pntd.0007675.s007.docx]

Dear Editors and Reviewers:

Thank you for your insightful comments and suggestions regarding our manuscript "Polarized Lung Inflammation and Tie2/Angiopoietin-Mediated Endothelial Dysfunction during Severe *Orientia tsutsugamushi* Infection" (#PNTD-D-19-01266) for consideration by *PLOS Neglected Tropical Diseases*. We have carefully revised this manuscript and would like to provide the point-by-point responses, accordingly.

**Reviewer #1**:
1. *“For Fig.6, the results of M0 macrophages (grown in media with neither LPS nor IL-4) must be included to compare with those of M1 and M2 macrophages. If the results of M0 macrophages are comparable to those of M2, the correct interpretation in discussion should be ‘it is possible that M1 macrophages are activated to more effectively kill OT in phagosome before its escape into the cytosol’.”*

We agree with the reviewer that M0 macrophages should be included in Figure 6. We have added new data for both qPCR and IFA analyses (see revised Figure 6, lines 268-274).

2. *“The detection of OT DNA by PCR cannot discriminate between live (in cytosol) and dead organisms (mostly in phagosome). In my experience, dead OT in macrophages can be detected by PCR for a few days. To discriminate live and dead bacteria, it is better to use confocal microscopy to study colocalization of OT and phagosome marker (e.g. LAMP1). OT is still colocalized in phagosome at 1h, but the colocalization of live OT in phagosome will decrease or disappear at 2h. In contrast, dead OT should longer persist in phagosome. Likewise, bacterial viability dyes may be tried. If the imaging experiments cannot be performed, the authors should carefully interpret the findings of bacterial load in M0, M1 and M2 macrophages.”*

We thank the reviewer for this good suggestion. We agree that our bacterial load data did not reveal the viability of OT. Currently, we do not have any data to discriminate live and dead OT. It was possible that, while dead/non-viable OT may make up a portion of the bacteria detected at our early time point (3 hpi), most, if not all, bacteria detect by 48 and 72 hpi, were viable and replicating. Further, given that we observed significant differences at later time points among M0, M1, and M2 macrophage groups, we believe that the differences we demonstrated in Figure 6 were due to bacterial replication rather than residual dead bacteria in phagosomes.

3. *“In fig.6B, since it was pure macrophage culture, why did you need to stain the cells with anti-IBA-1? A phagosome marker will be more meaningful. “*

Our macrophages were generated from bone marrow cells. Although the differentiation of bone marrow cells by using M-CSF results in high purity of BMDM, we would like to use IBA-1 to further exclude the contaminated cells. We currently do not have any data about phagosome markers; but we would like to keep this suggestion in mind for future and follow-up studies.

4. *“According to the upper comments, discussion (line 342-364) should be re-arranged and better summarized. The authors can discuss that TLR signaling and cytokines (e.g. TNF) from M1 macrophages can prime the cells to kill bacteria in phagosomes before cytosolic escape because these functions of M1 are well established in other intracellular pathogen (e.g. Listeria or Mycobacterium). In addition, the killing of cytosolic bacteria at later time by autophagy or other mechanisms by M1 macrophage can be mentioned and included in the discussion. Throughout the discussion, the authors should have compared these findings with those of other organisms that cause lung pathology such as Rickettsia spp. or Francisella tularensis or virus that causes pneumonia after hematogenous spread.”*

We have revised our discussion section according to the reviewer’s suggestion. We have addressed the importance of macrophage polarization in combating related intracellular bacterial pathogens (lines 347-351). Additionally, we have briefly mentioned a recent publication regarding macrophage autophagy during *O. tsutsugamushi* infection (lines 360-363), highlighting future autophagy-related research in the context of macrophage polarization.

5. *“Fig.1A Was the picture clear enough to suggest that OT infects and replicates in endothelial cells? If yes, please clearly specify the finding in the text in order to clearly explain the term ‘close association’ (line156-167).  On the other hand, the finding in Fig.1D did not explain the discussion line 301 ‘VEGFR on the infected ECs’.*

We have revised our discussion section according to the reviewer’s suggestion. The term “close association” (lines 157-158) in regards to the ICAM-1/*O. tsutsugamushi* staining was removed for clarity. We have also removed our VEGFR2 endothelial cell data, as it may be somewhat confusing to the reader and more pertinent in a future endothelial function study of infection.

*Please discuss and cite the references about infection of OT in ECs of visceral organs in mouse models.*

Information regarding previous identification of pulmonary endothelium as a target for *O. tsutsugamushi* infection was added to the **Introduction** section (lines 99-100), with the addition of our recently published review that highlights the shortcomings in knowledge of endothelial infection during scrub typhus (Ref #21: Scrub Typhus Pathogenesis: Innate Immune Response and Lung Injury During *Orientia tsutsugamushi* Infection. Front Microbiol Sep 6; 10:2065. Sept 2019).

*In addition, I noticed the upregulation of ICAM-1 on uninfected cells in Fig.1A. The role of secreted cytokines such as TNF in the activation of nearby ECs may be discussed to explain this finding.”*

To correlate our endothelial activation findings, we have also added information regarding the production of inflammatory cytokines (and their citations), which are known to activate the endothelium, including TNFα and IL-6, during scrub typhus (lines 298-302).

6. *“For Fig.4 page 48, Fig.4A the right flow cytometry gating strategy, the term M1 and M2 should be included in each quadrant in the figure. Few cells are CD80+ CD206+ double positive cells. What are the phenotype of these cells? On figure legend line 898 page 42, M1 should be CD80+CD206-? M2 cells are CD206+, CD80 negative or positive or regardless of CD80 expression?”*

We thank the reviewer for this point. We have added the M1 and M2 labels in the flow cytometry figure. Our data show that most of macrophages in the lung were M1-like (CD80+CD206-) cells, but there were very few of M2 (CD206+CD80-) cells. There was a small population of M1/M2 mixed cells (CD80+CD206+), which have been reported in human lung disease and may play a pathogenic role in the lung (ref: A circulating cell population showing both M1 and M2 monocyte/macrophage surface markers characterizes systemic sclerosis patients with lung involvement. Trombetta AC, *et al., Respir Res*. 2018; 19: 186). At this stage, we do not have any data for CD206+CD80- or CD206+CD80+ cells; however, we will keep this subpopulation of cells in mind for our future studies in lung- or liver-derived cells in our animal models.

7. *“Fig.4B page48 the word ‘activated’ is absent in the graph title.”*

We have revised this.

8. *“Fig.S3B Why did you use CD40 instead of CD80 staining for M1? The references of mouse M1 and M2 markers must be cited on the first appearance in the text.”*

M1 macrophages express M1-specific markers including CD40, CD80, and CD86, whereas M2 macrophages express M2-specific markers such as CD163, CD200R, and CD206. We have added a new reference for our choices in selecting the M1/M2 markers (lines 245-248, 982).

**Reviewer #2**: (No Response)

**Reviewer #3**:
1. *“Have the authors considered the potential impact of prior conditions such as malnutrition on the endothelium? Could these factors play synergistic roles with the infection? Mice models of malnutrition could help to elucidate this aspect. The authors may want to discuss on this.”*

This is an interesting point. Although we did not focus on malnutrition in our model in this manuscript, the malnutrition and endothelial activation/dysfunction is tightly linked (ref: NADPH oxidase and enhanced superoxide generation in intrauterine undernourished rats: involvement of the renin-angiotensin system. MC Franco, et al. Cardiovasc Res. 1;59(3):767-75. Sep 2003). While malnutrition is outside the scope of this paper, we recognize the importance of diet/metabolism in many immune cell populations and have mentioned related studies (ref: Impact of Childhood Malnutrition on Host Defense and Infection. MK Ibrahim et al. Clin Microbiol Rev. 30(4):919-971. Oct 2017). In our upcoming study of neutrophils during *O. tsutsugamushi* infection, we will need to account for malnutrition effects as mice lose weight during infection.

2. *“Between those markers of endothelial dysfunction, bioadrenomedullin is of particular interest. Intravascular bioadrenomedullin preserves endothelial integrity. There is an antibody, adrecizumab, which increases intravascular availability of bioadrenomedullin. It could be interesting to profile this marker if the authors have available serum. Nonetheless, absence of this data do not invalidate the interest of this study.”*

We thank the reviewer for this thoughtful suggestion. Unfortunately, we do not have serum samples from the current experiments. We will consider this new direction in the future.

3. *“In the timeline, what is first, endothelial dysfunction or M1 polarization leading to pro-inflammatory responses? This could help to clarify what is the trigger event of lung damage in this infection.”*

We agree with the reviewer that defining the early/first time of endothelial dysfunction or M1 polarization leading to proinflammatory responses is interesting and important. However, we’d like to examine the trigger event of lung damage in a separate report (via using different infection doses and side-by-side comparison with wild-type and gene-targeted knockout mice at multiple time points during the infection). To strengthen this manuscript, we have added a few lines in the **Discussion section**, implying our speculated timeline of “endothelial dysfunction or M1 polarization leading to pro-inflammatory responses.” (lines 335-339)

4. *“Do the authors have any data on Neutrophil degranulation proteins, such as MPO, MMPs, lactoferrin, or NGAL? It could be interested to evaluate their association and timing with endothelial dysfunction.”*

As suggested by the reviewer, we have included data on neutrophil activation/effector proteins. As shown in new **Figure S2**, our MPO data have indicated that endothelial stress marker increase (Ang2) correlates with increased MPO release, with observed colocalization of the two markers by D6-D9 of infection (lines 198-200).

5. *“Line 152 – The abbreviation of i.v should first appear here than in the line 418.”*

We have modified this.

6. *“Line 545- South Korea is an endemic country for scrub typhus and the baseline titers varies from region to region. Previously, the single cut off titers used have ranged from 1:10 to 1:400. How did the authors decide on ≥1:160 is a sufficient cut off titer on single serum/plasma specimen? It would be apt to cite a reference here.”*

This is a valid concern. The IgM titer was recommended as a diagnostic standard of scrub typhus in South Korea by Korean CDC, based on their serosurvey study of Korean patients (ref: A Serosurvey of *Orientia Tsutsugamushi* From Patients With Scrub Typhus. DM Kim et al.  Clin Microbiol Infect 16 (5), 447-51.  May 2010). This explanation has been added to the **Materials and Methods** section (lines 550-551).

7. *“Line 548 – healthy volunteer’s sera was negative at 1:160 dilution?”*

Yes. All the sera from healthy volunteers were negative for IgM at 1:160 dilution.
